# Assessment of technological level of stem cell research using principal component analysis

**DOI:** 10.1186/s40064-016-2494-9

**Published:** 2016-06-24

**Authors:** Sung Do Cho, Byung Hwan Hyun, Jae Kyeom Kim

**Affiliations:** Science and Technology Management Policy, University of Science and Technology, Daejeon, 305-350 Republic of Korea; Department of Business Consulting, Graduate School, Daejeon University, Daejeon, 300-716 Republic of Korea; School of Human Environmental Sciences, University of Arkansas, Fayetteville, AR 72701 USA

**Keywords:** Technology level assessment, Principal component analysis, Analysis of scientific literatures and patents, Stem cell

## Abstract

**Background:**

In general, technological levels have been assessed based on specialist’s opinion through the methods such as Delphi. But in such cases, results could be significantly biased per study design and individual expert.

**Findings:**

In this study, therefore scientific literatures and patents were selected by means of analytic indexes for statistic approach and technical assessment of stem cell fields. The analytic indexes, numbers and impact indexes of scientific literatures and patents, were weighted based on principal component analysis, and then, were summated into the single value. Technological obsolescence was calculated through the cited half-life of patents issued by the United States Patents and Trademark Office and was reflected in technological level assessment. As results, ranks of each nation’s in reference to the technology level were rated by the proposed method. Furthermore we were able to evaluate strengthens and weaknesses thereof.

**Conclusions:**

Although our empirical research presents faithful results, in the further study, there is a need to compare the existing methods and the suggested method.

## Background

Over the last 15 years, our knowledge of stem cell biology has exponentially increased. The result of that there is an ever-increasing array of stem cells such as adult stem cell (ASC), embryonic stem cell (ESC), and induced pluripotent stem cell (iPSC). In the early stage of stem cell researches, ASC and ESC were extensively investigated but iPSC has become one of the major stem cell research fields since the development of iPSC by the Shinya Yamanaka group in 2007 (Litterman and Ekins 2015). In 2012, even its infancy, the key scientists of iPSC shared the award of Nobel Prize in Physiology and Medicine, emphasizing the significant of the advent of reprogramming and cell fate conversion. Further, scientists have become actively involved in stem cell biology and translation, participating in new fundamental discoveries and leading efforts into applications in biotechnology and medicine. Thus, the technical assessment of stem cells including this study might be timely and warranted.

Various studies are underway on establishing methodologies for strategic research and development (R&D) planning, and there has been an increase in application of technological level assessment. The technological level assessment is intended to compare scientific or industrial technologies of countries, industries and/or companies (Han et al. 2010). And, a technological level, an object of assessment, can be defined as performance of a specific technology measured at a specific time point. In this, the assessment needs a comparison between either two technologies or two different time points in reference to technological capability.

In the present study, we assessed the technology level of stem cell research based on published scientific literatures and patent analysis at national levels. Specifically, we analyzed ASC, ESC, and iPSC for eleven nations: Canada (CA), China (CN), France (FR), Germany (DE), Israel (IL), Japan (JP), Singapore (SG), South Korea (KR), Taiwan (TW), United Kingdom (UK), and United States (US).

## Data collection

### Scientific literatures and patents for assessment of technological levels

Technological levels have been often assessed through specialized agencies. For example, in the US, the Office of Science and Technology Policy (OSTP) has been taking a primary role in assessment of nation’s technological levels. In JP, they conduct the technological level assessment prior to laying a science and technology master plan, wherewith it quantitatively and qualitatively assesses the levels of core technologies. In addition, the Japan Science and Technology Agency–Center for Research and Development Strategy (JST–CRDS) has been conducting international comparative assessment of technology level to support effective R&D policy-making since 2008. In KR, the levels of technologies (e.g., green technologies, information technologies, and industrial technologies) are often assessed by national agencies such as Korea Institute of Science and Technology Evaluation and Planning (KISTEP). The Table 1 depicts the summary of the recent cases of technology level assessment in different countries.

**Table 1:** Technology level assessments implemented in the US, Japan, and Korea

Country	Implementing agency	Assessment contents	Method	References
US	Office of Science Technology Policy (OSTP)	Subareas of 90 important national technologies Comparison of technological level against Japan and European countries	Likert-scale	(OSTP 1995)
	World Technology Evaluation Center (WTEC)	Physics and engineering Advances in life sciences and oncology Systems engineering for clean and renewable energy manufacturing	Experts review	(WTEC 2014) (WTEC 2013)
Japan	Japan Science and Technology Agency–Center for Research and Development Strategy (JST–CRDS)	Focus on 5 major areas of science and technology Assessment of basic research level (universities and institutes), applied research level (corporations), and industry competitiveness	Analysis of scientific literatures and patents (quantitative) Experts review(qualitative)	(CRDS 2013)
Korea	Korea Institute of Science Technology and Evaluation Planning (KISTEP)	Focused on 120 key science and technologies for the 3rd Science and Technology Basic Plan (2013–2017) Technology level and time gap of major 5 nations relative to the nation of the highest level	Analysis of scientific literatures and patents (quantitative) Delphi survey (qualitative)	(KISTEP 2013)

Patents are often regarded as one of the most representative technological outputs of R&D activities hence patents statistics might be a valuable source of information for technological strategy planning. It seems obvious therefore that patents statistics has been receiving more attention for implementing successful R&D as well as activities in high-tech industries such as biotechnology and information and communication technology. The Table 2 summarizes an important set of statistics used to analyze technological strategy in previous studies. The statistics were proposed for the evaluation of competitive positions in: number of patent, patent impact index, and cited-patent life time.

**Table 2:** Patent statistics for technology assessment in previous studies

Previous studies	Patent statistics		
	Number of patents	Patent impact index	Cited-patent life time
Huang et al. (2003)	0	0	
Ernst (2003)	0		
CHI Research Inc. (2005)	0	0	
Chia (2004)	0	0	
Yoo et al. (2006)			0
Cho et al. (2013)	0	0	
Cho and Park (2015)	0		

On the other hand, published research literatures could be also utilized as an excellent indicator for technology level assessment (Garfield and Welljams-Dorof 1992). Even though, numbers of scientific literatures have traditionally been regarded as an indicator of the productivity, quality of these works should not be overlooked. Such qualitative analysis however, is mostly performed by experts in the field thus is an often time-consuming and expensive process. In order to overcome these limitations, advent of citation databases, which track how often articles are referenced in subsequent publications, and by whom, have created new tools for indicating the impact of primary research literatures. By aggregating citation data, it is then possible to indicate the relative impact of individuals, journals, departments, institutions, and nations. In addition, the citation data can be used to identify emerging specialties, new technologies and even the structure of various research disciplines, fields, and science as a whole.

### Obsolescence rate

A technology becomes obsolescent over time. It might have been an up-to-date technology at one time, yet it may be regarded as an obsolete technology later. Bosworth (1978) analyzed statistical data on knowledge lie, from the creation of new technologies to their obsoleteness, by using the residual materials (Bosworth 1978). The usage rate of new technologies rose gradually and reached the peak on the 6th year after the creation of new technology, and then it showed a slow decline and reached nearly zero on the 16th year (Bosworth 1978). In the case of the manufacturing industry, for instance in UK, the obsolescence rate of technological knowledge estimated with renewed patents, reached approximately 10 %. In JP, the obsolescence rate was calculated from the reciprocal of average life of each industry, on the premise that the technological knowledge stock would decrease equally every year (KISTEP 2010). In other study, the obsolescence rate of R&D stock was estimated at 20 % because just 20 % of initial R&D expenses were developed into R&D stock (Pattel and Soete 1988). In this study, the obsolescence rate was estimated by the use of cited half-life (CHL). The CHL is intended to show how long specific journals or patents have been cited; more precisely, the period during which cumulative citation frequency reaches 50 %. In the study, therefore, the CHL was calculated using formula used in the Journal Citation Reports.

### Approaches for technological level assessment: current status

Technological level assessment has been conducted by different assessors for various technologies, yet through similar methods, whereupon it has the following limitations. First, technological level was assessed via qualitative methods such as Delphi or the focus group interview. Although the qualitative methods are easy to conduct, often these methods are susceptible to methodological approaches, per se (KISTEP 2011). Consequently, results may be impacted per how an experimental group is composed. Second, the existing quantitative assessments, using scientific literatures and patent statistics, did not compute technological obsolescence caused by lapse of time. In practice, however, a technology has been obsolescent over time and become less valuable in the end, warranting the novel definition to account for this. In the current study, therefore this technological obsolescence was quantitatively computed utilizing the CHL (i.e., the period during which cumulative citation frequency reaches 50 %), and then, reflected for the assessment. Further, to compensate erstwhile defects, this study focused on technological scientific literatures and patents to which bibliometrics can be applied. The composite index of technological levels was calculated with literatures and patent indexes, weighted through principal component analysis (PCA) whilst technological obsolescence was assessed through the term, CHL.

### Data collection

**Fig. 1:**
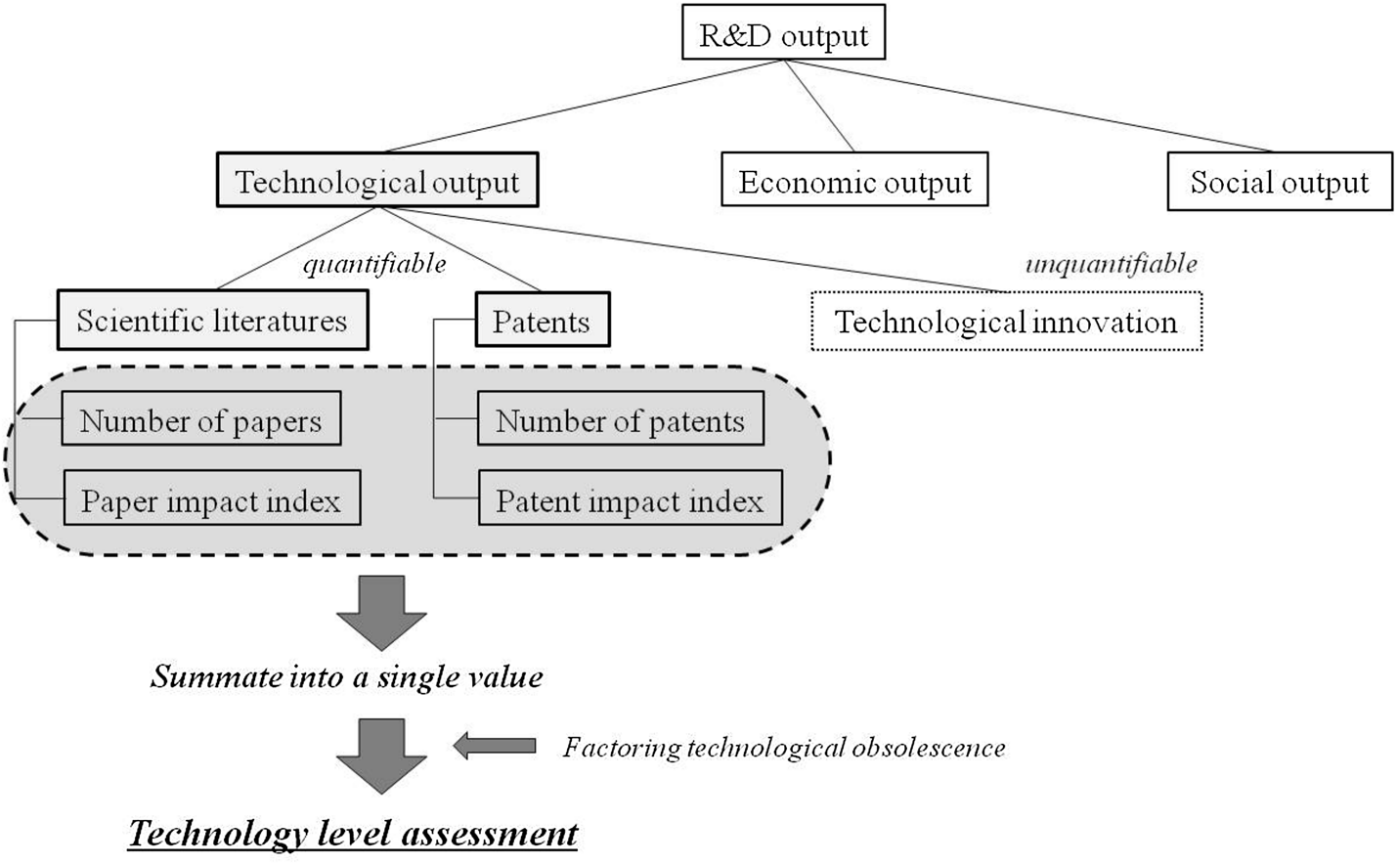
Assessment of technological level using quantifiable outputs, scientific literature and patent: a research model

In the present study, the Focust database was utilized for patents, respectively. For the database for scientific literature, we retrieved the articles using the database, Scopus, was utilized since it lists the largest numbers of journals (i.e., 21,568 journals) compared to other databases (e.g., Science Citation Index: 3746 journals; Arts and Humanities Citation Index: 1774 journals). Specific searching conditions were described in the Table 3. In addition, Table 4 shows specific indicators utilized for the analysis. Quantitative indicators were measured with the numbers of patents issued by the United States Patents and Trademark Office (USPTO) and scientific literatures listed in the database (i.e., Scopus). Moreover, qualitative indicators were measured using ‘Patent Impact Index’ and ‘Paper Impact Index.’ Such indicators were calculated in relation to every country and year. Every indicator was divided by the maximum value of every year, and the indicator values were re-scaled between 0 and 1 (Fig. 1).

**Table 3:** Variables used for scientific literature and patent searching

Variables	Definition
Scientific literature	
Database	Scopus
Document type	Primary research articles
Subject areas	Life sciences and health sciences
Patent	
Database	Focust
Patent type	Patents issued by the United States Patents and Trademark Office
Search formula	
Adult stem cell	“Adult stem cell*” or [(hematopoietic or neural or mesenchymal or intestinal or pancreatic or retinal or Epidermal or “umbilical cord blood” or mammary or endothelial or olfactory or “dental pulp derive”) and “stem cell*”]
Embryonic stem cell	“Embryonic stem cell*”
Induced pluripotent stem cell	“Induced pluripotent stem cell*” or (iPS and “stem cell*”)
Miscellaneous conditions	
Date range	Jan-01-1993–Dec-31-2012 (20 years)
Country	Canada, China, France, Germany, Israel, Japan, Singapore, Korea, Taiwan, United Kingdom, United States

Asterisk (*) was used as a placeholder for unknown or wildcard terms

**Table 4:** Indicators for scientific literatures and patent analysis

Indicators	Description	Calculation	
Number of patents	Quantity of patents	The number of patents issued by the United States Patents and Trademark Office	
Patent Impact Index	Quality of patents	${\text{PII}}_{\text{a}} = \frac{{\frac{{{\text{C}}_{\text{a}} }}{{{\text{N}}_{\text{a}} }}}}{{\frac{{{\text{C}}_{\text{t}} }}{{{\text{N}}_{\text{t}} }}}}$	Ca = the number of forward citations of patent in nation a Na = the number of patent in nation a Ct = the number of total forward citations of patents Nt = the number of total patents
Number of papers	Quantity of scientific literatures	The number of scientific literatures published by the Scopus	
Paper Impact Index	Quality of scientific literatures	${\text{PII}}_{\text{a}} = \frac{{\frac{{{\text{C}}_{\text{a}} }}{{{\text{N}}_{\text{a}} }}}}{{\frac{{{\text{C}}_{\text{t}} }}{{{\text{N}}_{\text{t}} }}}}$	Ca = the number of forward citations of scientific literature in nation a Na = the number of scientific literatures in nation a Ct = the number of total forward citations of scientific literatures Nt = the number of total scientific literatures

### Statistical analysis

The PCA was utilized to reduce correlated multivariate data to low-dimensional data in order to prevent the loss of information, meaning simplifying complex data sets for better interpretation (Raychaudhuri et al. 2000). PCs were generated via the summation of weighted variables. In this process, the weighting was determined via the optimization of information contained in initial variables to the end which may represent the widest dispersion. In case the initial variable contains a large number of principal components, every component should not be correlated, in other words, the inter-variable covariance should be 0. The relational expression was expressed in the Eq. 1 as follow:

1 $$\begin{aligned} PC_{1}& = a_{11} x_{1} + a_{12} x_{2} + \cdots + a_{1n} x_{n} \hfill \\ PC_{2} &= a_{21} x_{1} + a_{22} x_{2} + \cdots + a_{2n} x_{n} \hfill \\& \ldots \hfill \\ PC_{m}& = a_{m1} x_{1} + a_{m2} x_{2} + \cdots + a_{mn} x_{n} \hfill \\ \end{aligned}$$

In the formula, PC_1_ to PC_m_ represent principal components while *x*_*1*_ to *x*_*n*_ indicate initial variables; *a*_11_ to *a*_*mn*_ mean weightings. The PCs were selected when its eigenvalue is 1 or higher (according to the Kaiser’s rule). Weightings, regarding respective variables, could be calculated by the reverse use of the factor matrix between PCs and variables. In this study, the composite technological level was analyzed through the weightings figured out with respect to each variable.

Lastly, in order to assess the technical level of stem cell technology, four weightings obtained from PCA were summated into a single value, from which technological level index was calculated. Then, CHL was applied to the technological obsolescence rate. With the technological level index, technological levels were analyzed in relation to years and countries. The formula to assess the technology level is described as below:

2 $$\begin{aligned} TL_{t} & = \mathop \sum \limits_{{t^{\prime}}}^{t} \left( {\left( {NT_{t} \times NT_{\omega } } \right) + \left( {PT_{t} \times PT_{\omega } } \right) \times \left( {NR_{t} \times NR_{\omega } } \right) \times \left( {PR_{t} \times PR_{\omega } } \right)} \right) \times \delta_{t} \\ TL_{t} & = Technology \, level \, in \, t \, year, \, t^{\prime} = \, initial \, year, \\ NT \, & = NP\_Patent \, \left( {the \, number \, of \, patent} \right), \, NT_{\omega } = \, weight \, of \, NT \\ PT & = PII\_Patent \, \left( {patent \, impact \, index} \right), \, PT_{\omega } = \, weight \, of \, PT \\ NR & = NP\_Paper \, \left( {the \, number \, of \, paper} \right), \, NR_{\omega } = \, weight \, of \, NR \\ PR & = PII\_Paper \, \left( {paper \, impact \, index} \right), \, PR_{\omega } = \, weight \, of \, PR \\ \delta & = Obsolescence \, rate \\ \end{aligned}$$

## Results and discussion

### Analysis of scientific literatures and patents

Stem cell-related scientific literatures and patents (e.g., ASC, ESC, and iPS cells) were retrieved, and then, the NP and PII were analyzed in relation to years and countries (Table 5). The indexes of different units were divided by the maximum value of each year, and all of them re-scaled between 0 and 1 (Table 6).

**Table 5:** Results of scientific literatures and patent analysis

Indicators	Year	CA	CN	DE	FR	UK	IL	JP	KR	SG	TW	US
Adult stem cell												
NP_Patent	1993	1	n/a	n/a	n/a	n/a	n/a	n/a	n/a	n/a	n/a	3
	⋮	⋮	⋮	⋮	⋮	⋮	⋮	⋮	⋮	⋮	⋮	⋮
	2012	3	n/a	3	4	3	6	8	4	6	4	79
PII_Patent	1993	0.42	n/a	n/a	n/a	n/a	n/a	n/a	n/a	n/a	n/a	1.19
	⋮	⋮	⋮	⋮	⋮	⋮	⋮	⋮	⋮	⋮	⋮	⋮
	2012	0.36	n/a	n/a	2.96	n/a	0.18	2.22	n/a	0.81	n/a	1.06
NP_Paper	1993	1	n/a	5	4	4	1	6	n/a	n/a	n/a	37
	⋮	⋮	⋮	⋮	⋮	⋮	⋮	⋮	⋮	⋮	⋮	⋮
	2012	80	747	169	105	137	33	194	198	30	64	694
PII_Paper	1993	0.58	n/a	1.01	0.74	2.77	0.60	0.50	n/a	n/a	n/a	0.94
	⋮	⋮	⋮	⋮	⋮	⋮	⋮	⋮	⋮	⋮	⋮	⋮
	2012	1.04	0.49	1.32	1.12	1.56	0.95	0.88	0.87	1.32	0.77	1.42
Embryonic stem cell												
NP_Patent	1993	n/a	n/a	n/a	n/a	n/a	n/a	n/a	n/a	n/a	n/a	n/a
	⋮	⋮	⋮	⋮	⋮	⋮	⋮	⋮	⋮	⋮	⋮	⋮
	2012	1	n/a	n/a	1	3	2	10	2	3	1	55
PII_Patent	1993	n/a	n/a	n/a	n/a	n/a	n/a	n/a	n/a	n/a	n/a	n/a
	⋮	⋮	⋮	⋮	⋮	⋮	⋮	⋮	⋮	⋮	⋮	⋮
	2012	n/a	n/a	n/a	n/a	n/a	n/a	1.99	n/a	0.28	n/a	1.04
NP_Paper	1993	2	n/a	6	1	4	1	2	n/a	n/a	n/a	14
	⋮	⋮	⋮	⋮	⋮	⋮	⋮	⋮	⋮	⋮	⋮	⋮
	2012	31	109	54	25	67	18	56	44	20	10	261
PII_Paper	1993	6.43	n/a	0.85	0.02	0.10	0.37	0.15	n/a	n/a	n/a	0.78
	⋮	⋮	⋮	⋮	⋮	⋮	⋮	⋮	⋮	⋮	⋮	⋮
	2012	0.95	0.70	0.93	1.17	1.23	0.60	0.86	0.51	0.87	0.34	1.25
Induced pluripotent stem cell												
NP_Patent	1993	n/a	n/a	n/a	n/a	n/a	n/a	n/a	n/a	n/a	n/a	n/a
	⋮	⋮	⋮	⋮	⋮	⋮	⋮	⋮	⋮	⋮	⋮	⋮
	2012	n/a	n/a	n/a	n/a	n/a	n/a	4	n/a	n/a	1	2
PII_Patent	1993	n/a	n/a	n/a	n/a	n/a	n/a	n/a	n/a	n/a	n/a	n/a
	⋮	⋮	⋮	⋮	⋮	⋮	⋮	⋮	⋮	⋮	⋮	⋮
	2012	n/a	n/a	n/a	n/a	n/a	n/a	1.75	n/a	n/a	n/a	n/a
NP_Paper	1993	n/a	n/a	n/a	n/a	n/a	n/a	n/a	n/a	n/a	n/a	n/a
	⋮	⋮	⋮	⋮	⋮	⋮	⋮	⋮	⋮	⋮	⋮	⋮
	2012	7	51	18	9	12	6	63	7	1	11	111
PII_Paper	1993	n/a	n/a	n/a	n/a	n/a	n/a	n/a	n/a	n/a	n/a	n/a
	⋮	⋮	⋮	⋮	⋮	⋮	⋮	⋮	⋮	⋮	⋮	⋮
	2012	1.64	0.66	0.81	1.76	1.86	1.29	0.69	0.84	0.62	1.07	1.16

*CA* Canada, *CN* China, *FR* France, *DE* Germany, *IL* Israel, *JP* Japan, *SG* Singapore, *KR* Korea, *TW* Taiwan, *UK* United Kingdom, *US* United States, *NP_Patent* number of patents, *PII_Patent* Patent Impact Index, *NP_Paper* Number of papers, *PII_Paper* Paper Impact Index

**Table 6:** Re-scaled indexes

Indicators	Year	CA	CN	DE	FR	UK	IL	JP	KR	SG	TW	US
Adult stem cell												
NP_Patent	1993	0.33^a^	n/a	n/a	n/a	n/a	n/a	n/a	n/a	n/a	n/a	1.00
	⋮	⋮	⋮	⋮	⋮	⋮	⋮	⋮	⋮	⋮	⋮	⋮
	2012	0.04	n/a	0.04	0.05	0.04	0.08	0.10	0.05	0.08	0.05	1.00
PII_Patent	1993	0.35	n/a	n/a	n/a	n/a	n/a	n/a	n/a	n/a	n/a	1.00
	⋮	⋮	⋮	⋮	⋮	⋮	⋮	⋮	⋮	⋮	⋮	⋮
	2012	0.12	n/a	n/a	1.00	n/a	0.06	0.75	n/a	0.27	n/a	0.36
NP_Paper	1993	0.03	n/a	0.14	0.11	0.11	0.03	0.16	n/a	n/a	n/a	1.00
	⋮	⋮	⋮	⋮	⋮	⋮	⋮	⋮	⋮	⋮	⋮	⋮
	2012	0.11	1.00	0.23	0.14	0.18	0.04	0.26	0.27	0.04	0.09	0.93
PII_Paper	1993	0.09	n/a	0.16	0.12	0.43	0.09	0.08	n/a	n/a	n/a	0.15
	⋮	⋮	⋮	⋮	⋮	⋮	⋮	⋮	⋮	⋮	⋮	⋮
	2012	0.56	0.26	0.71	0.60	0.84	0.51	0.47	0.47	0.71	0.41	0.76
Embryonic stem cell												
NP_Patent	1993	n/a	n/a	n/a	n/a	n/a	n/a	n/a	n/a	n/a	n/a	n/a
	⋮	⋮	⋮	⋮	⋮	⋮	⋮	⋮	⋮	⋮	⋮	⋮
	2012	0.01	n/a	n/a	0.01	0.04	0.03	0.13	0.03	0.04	0.01	0.70
PII_Patent	1993	n/a	n/a	n/a	n/a	n/a	n/a	n/a	n/a	n/a	n/a	n/a
	⋮	⋮	⋮	⋮	⋮	⋮	⋮	⋮	⋮	⋮	⋮	⋮
	2012	n/a	n/a	n/a	n/a	n/a	n/a	0.67	n/a	0.09	n/a	0.35
NP_Paper	1993	0.05	n/a	0.16	0.03	0.11	0.03	0.05	n/a	n/a	n/a	0.38
	⋮	⋮	⋮	⋮	⋮	⋮	⋮	⋮	⋮	⋮	⋮	⋮
	2012	0.04	0.15	0.07	0.03	0.09	0.02	0.07	0.06	0.03	0.01	0.35
PII_Paper	1993	1.00	n/a	0.13	0.00	0.02	0.06	0.02	n/a	n/a	n/a	0.12
	⋮	⋮	⋮	⋮	⋮	⋮	⋮	⋮	⋮	⋮	⋮	⋮
	2012	0.51	0.37	0.50	0.63	0.66	0.32	0.46	0.28	0.47	0.18	0.67
Induced pluripotent stem cell												
NP_Patent	1993	n/a	n/a	n/a	n/a	n/a	n/a	n/a	n/a	n/a	n/a	n/a
	⋮	⋮	⋮	⋮	⋮	⋮	⋮	⋮	⋮	⋮	⋮	⋮
	2012	n/a	n/a	n/a	n/a	n/a	n/a	0.05	n/a	n/a	0.01	0.03
PII_Patent	1993	n/a	n/a	n/a	n/a	n/a	n/a	n/a	n/a	n/a	n/a	n/a
	⋮	⋮	⋮	⋮	⋮	⋮	⋮	⋮	⋮	⋮	⋮	⋮
	2012	n/a	n/a	n/a	n/a	n/a	n/a	0.59	n/a	n/a	n/a	n/a
NP_Paper	1993	n/a	n/a	n/a	n/a	n/a	n/a	n/a	n/a	n/a	n/a	n/a
	⋮	⋮	⋮	⋮	⋮	⋮	⋮	⋮	⋮	⋮	⋮	⋮
	2012	0.01	0.07	0.02	0.01	0.02	0.01	0.08	0.01	0.00	0.01	0.15
PII_Paper	1993	n/a	n/a	n/a	n/a	n/a	n/a	n/a	n/a	n/a	n/a	n/a
	⋮	⋮	⋮	⋮	⋮	⋮	⋮	⋮	⋮	⋮	⋮	⋮
	2012	0.88	0.35	0.44	0.94	1.00	0.69	0.37	0.45	0.33	0.58	0.62

*CA* Canada, *CN* China, *FR* France, *DE* Germany, *IL* Israel, *JP* Japan, *SG* Singapore, *KR* Korea, *TW* Taiwan, *UK* United Kingdom, *US* United States, *NP_Patent* number of patents, *PII_Patent* Patent Impact Index, *NP_Paper* number of papers, *PII_Paper* Paper Impact Index

^a^0.33 is calculated from the result of re-scaling; 1 (NP_Patent of CA in 1993)/3 (NP_Patent of US (maximum value) in 1993)

### PCA

In order to determine the weightings of indexes, PCA was conducted with respect to the indexes of published literatures and patents registered between 1993 and 2012. A total of 470 sets of data were used, exclusive of data of which values were 0 for four variables (Table 7). Then, calculations were made of four components, their eigenvalues and cumulative contribution rates. The eigenvalues of the PC1 and the PC2 were 2.145 and 1.028, respectively, and their contribution rates were 53.6 and 25.7 % respectively. These two PCs comprised of 79.3 % of the total data set (Table 8). Eigenvalues of the two PCs (Table 8) and component matrix (Table 9) were used to calculate the weightings of indexes (Table 10).

**Table 7:** Simple statistics of variables and their correlation coefficients

	N^a^	Mean	Standard deviation	NP_Patent	PII_Patent	NP_Paper	PII_Paper
NP_Patent	470	0.091	0.234	1.000			
PII_Patent	470	0.173	0.296	0.556	1.000		
NP_Paper	470	0.149	0.232	0.741	0.333	1.000	
PII_Paper	470	0.404	0.266	0.146	0.224	0.017	1.000

*NP_Patent* number of patents, *PII_Patent* Patent Impact Index, *NP_Paper* number of papers, *PII_Paper* Paper Impact Index

^a^20 (years) × 11 (countries) × 3 (adult stem cell, embryonic stem cell, iPS cell)—190 (the number of cases when four indicators (NP_Patent, PII_Patent, NP_Paper, PII_Paper) are all n/a) = 470

**Table 8:** Eigenvalue and cumulative variance of principal components (PCs)

PCs	Eigenvalue	Variance (%)	Cumulative variance (%)
PC1	2.15	53.62	53.62
PC2	1.03	25.70	79.33
PC3	0.62	15.42	94.75
PC4	0.21	5.25	100.00

PC1 and PC2 were selected as their eigenvalues are higher than 1

**Table 9:** Component matrix

	PC1	PC2
NP_Patent	0.924	−0.139
PII_Patent	0.740	0.232
NP_Paper	0.817	−0.359
PII_Paper	0.275	0.909

*NP_Patent* number of patents, *PII_Patent* Patent Impact Index, *NP_Paper* number of papers, *PII_Paper* Paper Impact Index

**Table 10:** Weights for statistics used in assessment of technological level

	Weight^a^	%
NP_Patent	1.838	27.97
PII_Patent	1.826	27.79
NP_Paper	1.384	21.06
PII_Paper	1.524	23.19

*NP_Patent* number of patents, *PII_Patent* Patent Impact Index, *NP_Paper* number of papers, *PII_Paper* Paper Impact Index

^a^Weights are calculated using Tables 6 and 7. The specific calculation is as follow: $$\left( {\begin{array}{*{20}c} {0.924} & { - 0.139} \\ {0.740} & {0.232} \\ {0.817} & { - 0.359} \\ {0.275} & {0.909} \\ \end{array} } \right)\left( {\begin{array}{*{20}c} {2.145} \\ {1.208} \\ \end{array} } \right) = \left( {\begin{array}{*{20}c} {1.838} \\ {1.826} \\ {1.384} \\ {1.524} \\ \end{array} } \right)$$

### Obsolescence rate

**Fig. 2:**
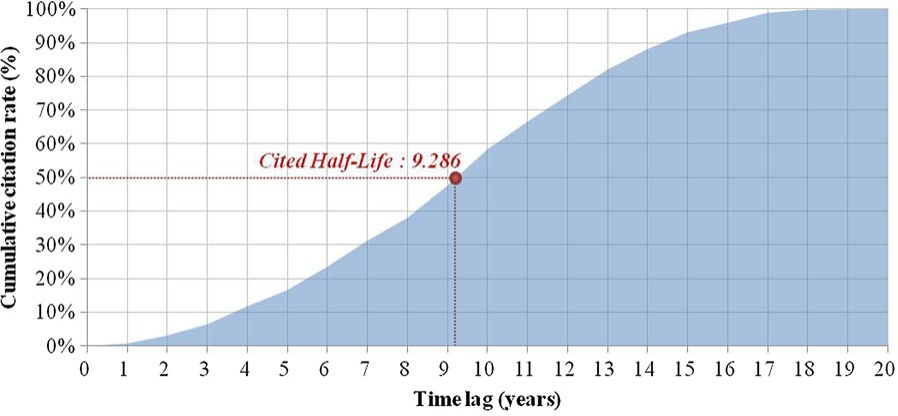
Cumulative citation frequency after patent issued for the fields of stem cell research

**Fig. 3:**
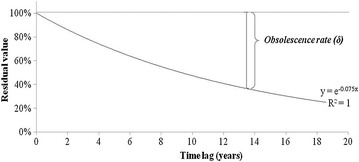
The obsolescence rate curve. The curve of obsolescence rate was created given the calculated CHL (9.286 years) which indicates that 50 % of the residual value would be decreased by every 9.286 years. The obsolescence rate at certain time point can be calculated via subtracting residual value out of 100 % (see Table 13 for the residual value)

The CHL was calculated using the citation frequency of USPTO issued patents in order to determine the technological obsolescence rate. In order to analyze the citation frequency, numbers of citations per each year were analyzed based on the year of registration of patents and expressed as percentile as shown in the Table 11. It indicates that the patent was citied 19.4 times on its 4th year from the point where it was registered if it was cited 100 times on the year of registration. In this, a calculation was made of the annual citation frequency of patents registered between 1993 and 2002. The Fig. 2 depicts the cumulative citation frequency of every year. In the result, CHL was 9.286 (Table 12; Fig. 2). Specifically, the patents averagely reached CHL 9.286 years after the registration. The Table 13 and Fig. 3 show the annual obsolescence rate on the premise that a technology obsolesces 50 % every 9.286 years.

**Table 11:** Percentage of citation frequency after patents issued

Issue year	Y + 0	Y + 1	Y + 2	Y + 3	Y + 4	Y + 5	Y + 6	Y + 7	Y + 8	Y + 9	Y + 10
1993	0.0	0.0	6.0	3.0	19.4	6.0	17.9	13.4	6.0	11.9	6.0
1994	0.0	2.9	0.0	5.7	2.9	0.0	5.7	8.6	2.9	2.9	5.7
1995	0.0	0.4	3.2	4.2	5.6	7.3	5.9	4.2	4.3	4.5	3.6
1996	0.1	0.4	2.5	3.0	3.4	4.8	5.6	5.4	4.3	4.4	5.2
1997	0.1	0.3	2.3	3.8	4.0	3.8	5.5	6.4	4.5	6.8	6.5
1998	0.0	0.8	1.9	2.5	3.1	4.7	4.3	4.3	7.1	5.7	8.5
1999	0.1	0.6	2.3	2.9	4.8	4.3	3.2	6.8	6.1	9.2	12.4
2000	0.0	0.8	2.1	4.4	4.9	6.2	6.0	6.7	8.4	12.9	15.9
2001	0.0	0.5	3.0	2.6	3.0	6.0	7.9	9.0	9.8	17.6	15.0
2002	0.0	0.8	0.5	1.0	2.5	4.8	7.3	12.3	14.5	20.0	28.7
Mean	0.0	0.8	2.4	3.3	5.3	4.8	6.9	7.7	6.8	9.6	10.7

Issue year	Y + 11	Y + 12	Y + 13	Y + 14	Y + 15	Y + 16	Y + 17	Y + 18	Y + 19	Y + 20	Total
1993	0.0	1.5	1.5	0.0	0.0	0.0	4.5	1.5	1.5	0.0	100.0
1994	0.0	8.6	14.3	8.6	11.4	2.9	11.4	5.7	0.0	–	100.0
1995	6.0	4.3	6.8	8.2	10.2	8.7	10.6	2.0	–	–	100.0
1996	5.1	7.8	7.9	11.5	11.6	13.9	3.3	–	–	–	100.0
1997	6.7	8.1	12.4	12.3	13.8	2.7	–	–	–	–	100.0
1998	10.6	13.3	13.6	16.8	2.9	–	–	–	–	–	100.0
1999	13.0	14.0	17.0	3.5	–	–	–	–	–	–	100.0
2000	12.7	15.7	3.2	–	–	–	–	–	–	–	100.0
2001	20.7	4.7	–	–	–	–	–	–	–	–	100.0
2002	7.5	–	–	–	–	–	–	–	–	–	100.0
Mean	8.2	7.8	7.7	6.1	5.0	2.8	3.0	0.9	0.1	0.0	100.0

**Table 12:** Calculation of cited half-life (CHL)

Time lag (years)	Citation frequency (%)	Cumulative citation frequency (%)
Y + 0	0.0	0.0
Y + 1	0.8	0.8
Y + 2	2.4	3.1
Y + 3	3.3	6.5
Y + 4	5.3	11.8
Y + 5	4.8	16.6
Y + 6	6.9	23.5
Y + 7	7.7	31.3
Y + 8	6.8	38.0
Y + 9	9.6	47.6
Y + 10	10.7	58.4
Y + 11	8.2	66.6
Y + 12	7.8	74.4
Y + 13	7.7	82.1
Y + 14	6.1	88.2
Y + 15	5.0	93.1
Y + 16	2.8	96.0
Y + 17	3.0	98.9
Y + 18	0.9	99.9
Y + 19	0.1	100.0
Y + 20	0.0	100.0
CHL = 9 + (50 − 47.6)/(58.4 − 50) = 9.286

**Table 13:** Obsolescence rate

Time lag (years)	Residual value (%)	Obsolescence rate (%)
0	100.0	0.0
1	92.8	7.2
2	86.1	13.9
3	79.9	20.1
4	74.1	25.9
5	68.7	31.3
6	63.8	36.2
7	59.2	40.8
8	54.9	45.1
9	50.9	49.1
11	43.8	56.2
12	40.7	59.3
13	37.7	62.3
14	35.0	65.0
15	32.5	67.5
16	30.1	69.9
17	27.9	72.1
18	25.9	74.1
19	24.1	75.9
20	20.7	79.3

### Assessment of technological level

**Fig. 4:**
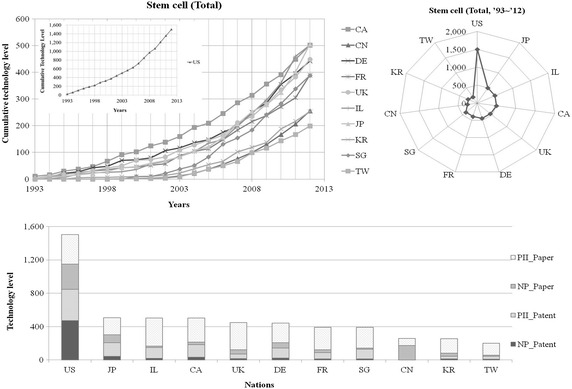
Technology level of stem cell (total)

The technological level, assessed at a specific point of time, means the technological stock calculated up to then, and thus the cumulative value was calculated for the assessment of annual technological levels. Prior to the calculation of the cumulative value, a time-series analysis was conducted with respect to the technological level, by the use of the annual obsolescence rate. The obsolescence rates shown in the Table 13 were reflected in the re-scaled indicators of scientific literatures and patents, and the levels of technologies presented every year were assessed at the present point in time (Fig. 4). As results, the US has the highest technological level, followed by JP, IL, CA, UK, DE, FR, SG, CN, KR and TW, respectively; the technological levels of Asian countries, except for JP, were relatively lower compared to those of European countries. In case of the US ranked first, 4 scientific literature and patent indicators showed high percentages without significant differences. In CN, however scientific literatures accounted for a high percentage of the technological level, whereas patents or patent impact indexes made up a very small part. IL and CA had high levels of NP but low levels of PII; overall, however their technological levels were relatively high.

**Fig. 5:**
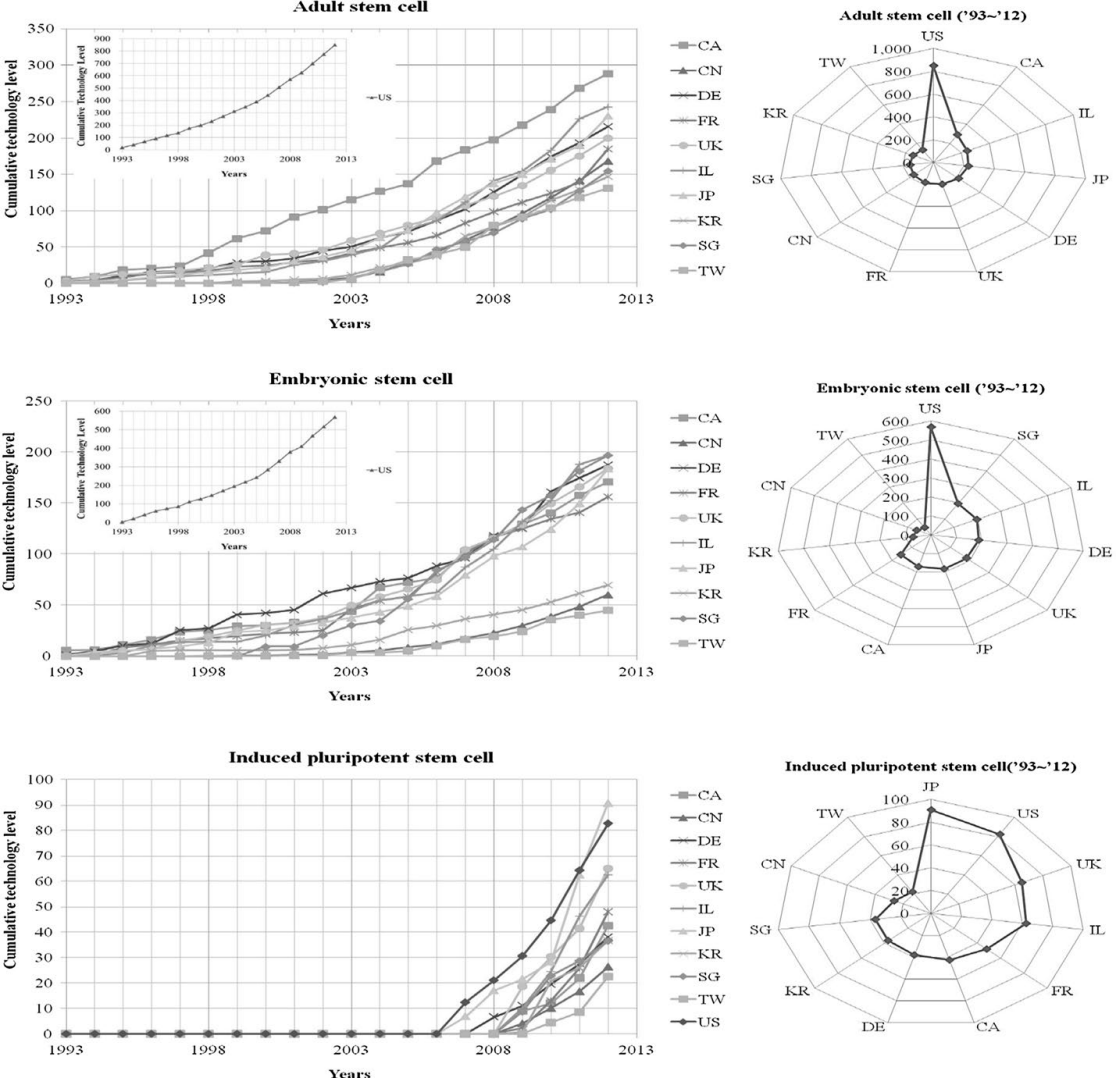
Technology level of stem cell (technological classification)

When it comes to the ASC, the US has the highest technological level, followed by CA, IL, JP and DE (Table 14; Fig. 5). In case of CA, the technological level has been rapidly improved, in comparison with other countries, since the late 1990s. Likewise, the US was rated first in the field of ESC and SG was the second, followed by IL and DE. When it comes to the ASC, countries did not show any significant difference, except for the US. In the field of ESC, SG, IL, DE, UK, JP, CA and FR constituted a group, while KR, CN and TW represented another group. There was remarkable difference between the two groups (Table 14; Fig. 5).

**Table 14:** Technological levels of countries in stem cell field

Cell type	Nation	NP_Patent	PII_Patent	NP_Paper	PII_Paper	Total
Adult stem cell	US	300.7 (1)	191.2 (1)	207.2 (1)	150.2 (1)	849.3 (1)
	CA	22.4 (3)	109.2 (2)	22.1 (8)	134.6 (4)	288.2 (2)
	IL	13.7 (5)	84.8 (3)	8.1 (10)	136.1 (3)	242.6 (3)
	JP	23.9 (2)	58.8 (4)	61.1 (3)	86.0 (8)	229.8 (4)
	DE	14.6 (4)	44.4 (6)	42.8 (4)	113.8 (6)	215.6 (5)
	UK	5.7 (9)	19.8 (10)	28.7 (5)	145.0 (2)	199.3 (6)
	FR	6.7 (7)	34.7 (7)	25.3 (7)	118.0 (5)	184.7 (7)
	CN	0.4 (11)	– (n/a)	142.3 (2)	25.5 (11)	168.2 (8)
	SG	6.5 (8)	48.6 (5)	5.3 (11)	93.9 (7)	154.4 (9)
	KR	8.6 (6)	26.9 (9)	27.1 (6)	84.0 (10)	146.7 (10)
	TW	4.8 (10)	31.4 (8)	9.8 (9)	84.8 (9)	130.7 (11)
Embryonic stem cell	US	170.2 (1)	183.1 (1)	83.5 (1)	131.7 (2)	568.6 (1)
	SG	4.0 (8)	69.3 (3)	5.7 (10)	117.8 (4)	196.8 (2)
	IL	5.5 (5)	44.6 (5)	6.8 (9)	139.8 (1)	196.7 (3)
	DE	5.4 (6)	74.6 (2)	20.4 (5)	87.3 (7)	187.7 (4)
	UK	10.4 (3)	34.7 (8)	20.5 (4)	118.5 (3)	184.1 (5)
	JP	14.9 (2)	64.0 (4)	28.0 (2)	77.0 (8)	183.9 (6)
	CA	9.5 (4)	39.7 (7)	9.5 (6)	112.0 (5)	170.7 (7)
	FR	4.8 (7)	40.3 (6)	9.4 (7)	101.8 (6)	156.3 (8)
	KR	2.6 (10)	5.5 (9)	8.6 (8)	53.4 (9)	70.0 (9)
	CN	1.2 (11)	1.5 (11)	21.9 (3)	35.8 (11)	60.4 (10)
	TW	2.7 (9)	3.0 (10)	2.0 (11)	37.5 (10)	45.2 (11)
Induced pluripotent stem cell	JP	1.9 (1)	42.2 (1)	4.7 (2)	41.9 (6)	90.7 (1)
	US	1.2 (2)	– (n/a)	10.0 (1)	71.3 (1)	82.6 (2)
	UK	– (n/a)	– (n/a)	1.0 (5)	64.0 (2)	65.0 (3)
	IL	– (n/a)	– (n/a)	0.7 (6)	61.8 (3)	62.6 (4)
	FR	– (n/a)	– (n/a)	0.6 (9)	47.4 (4)	48.0 (5)
	CA	– (n/a)	– (n/a)	0.5 (10)	42.1 (5)	42.5 (6)
	DE	– (n/a)	– (n/a)	1.6 (4)	36.5 (7)	38.1 (7)
	KR	– (n/a)	– (n/a)	0.7 (7)	36.0 (9)	36.7 (8)
	SG	– (n/a)	– (n/a)	0.3 (11)	36.1 (8)	36.5 (9)
	CN	– (n/a)	– (n/a)	3.4 (3)	22.9 (10)	26.3 (10)
	TW	0.4 (3)	– (n/a)	0.7 (8)	21.5 (11)	22.5 (11)

The number in parentheses indicates a ranking among countries

*NP_Patent* number of patents, *PII_Patent* Patent Impact Index, *NP_Paper* number of papers, *PII_Paper* Paper Impact Index

SG was found to be inferior to North American and European countries in the field of ASC as well as iPSC, yet in the ESC, it was the second highest country after the US. Of note, the technological level of human embryonic stem cell (hESC) was particularly high, hence, in a way to support the feasibility of the analysis result, we further reviewed the current hESC banks of each country (Korea Centers for Disease Control and Prevention, 2013). As expected, Singapore Stem Cell Consortium (SSCC) particularly focused on the hESC over other stem cell types; in fact it appeared that the SSCC developed clinical grade hESC in 2007 via their good manufacturing practice facilities. The Singapore Stem Cell Bank which is operated by the SSCC is currently selling four clinical grade hESC stocks as well (Korea Centers for Disease Control and Prevention 2013). It has been widely accepted that the development of clinical grade hESC requires cutting-edge technology which results only two countries have been successful so far (i.e., US and SG). To be specific, the US has developed two clinical grade cell stocks, while SG developed six clinical grade cell stocks (Crook et al. 2007). Given that, it seems reasonable to believe that the technological level of SG in the field of hESC stands out. The US stands unchallenged in the fields of ASC and ESC, while JP was the best country in the field of iPSC, over the US, which also makes sense given that (1) the Kyoto University was successful to generate the first iPSC in conjunction with the University of Wisconsin-Madison in 2007 and (2) JP produced a Nobel Prize winner in physiology or medicine, for iPSC in 2012.

The Table 14 summarizes rankings of technological levels of each stem cell field. First, not surprisingly, the US whose total technological level was the highest represented the highest values in four detail indicators (i.e., NP_Patent, PII_Patent, NP_Paper and PII_Paper) in the field of ASC. CA and IL whose total technological level was the second and third highest were relatively low in this field. Even though CN showed the second highest in number of scientific literatures (i.e., NP_Paper) after the US, it showed the lowest values in other indicators among the countries in comparison which partially explains their low ranking in the total technological level (8th out of 11 countries in the study; Table 14).

In the field of ESC, again, the US showed the highest values in all indicators except for PII_Paper, and its total technological level was highest. The scores of SG and IL in NP Patent and NP_Paper were low, but their values in PII_Patent and PII_Paper were relatively high which lead them to be placed in higher ranking in the field (2nd and 3rd out of 11 countries; Table 14). The technological level of CN in the field of ESC was the third highest in NP_Paper (alike in the field of ASC) after the US and JP, yet other indicators were (i.e., NP_Patent, PII_Patent, and PII_Paper) were the lowest amongst the countries.

On the other hand, due to the infancy of the field of the iPSC, the first patent was issued by the USPTO in 2011. Moreover, only three countries (US, JP, and TW) are holding the iPSC related patents issued by the USPTO. In this field, JP whose total score was the highest obtained a high value in patent-related indicators (NP_Patent and PII_Patent). The US showed the highest value in scientific literatures-related indicators (NP_Paper and PII_Paper), but they were ranked the second in the total technology level after the JP. If the new technology evaluation method presented in this study is applied, it is expected that not only the total technological level evaluation but also the comparison of individual indicators which compose the technological level will be possible.

This study assessed technological level utilizing a new quantitative method with a view to compensating limitations of the existing methods, i.e., qualitative assessment based on specialists’ opinion and quantitative one based on patent indicators. In this study, not only patents yet also scientific literatures were applied into the model as analytical indicators, the number of papers (NP_Paper), the paper impact index (PII_Paper), the number of patents (NP_Patent) and the patent impact index (PII_Patent) hence provide important insight for future R&D planning and establishment of related policy. There are two strengths of the assessment model we proposed herein compared to previous quantitative methods. First, a number of indexes for both scientific literatures and patents were summated into the single value based on the PCA. Although previous studies have attempted to summate a single value by analytic hierarchy process or specialists’ opinion (Cho and Park 2015), there was a limitation of multi-collinearity between indexes which may doubly affect overall assessment of technology levels; thus, the authors attempted to exclude multi-collinearity by using the PCA. Second, the existing quantitative assessments may not be appropriate to take account of technological obsolescence, because both recently registered and former patents were calculated as simple numeric values without consideration for technological obsolescence which made us to reflect it, caused by the lapse of time, in the technological level assessment. To achieve this, annual technological obsolescence was computed through the CHL. Nonetheless, in future, it is warranted to make a direct comparison between existing qualitative assessment methods against the present results as well as the methodological approach in terms of validity and reliability. Further, application of additional quantifiable indicators may result more accurate assessment as well. Lastly, it is required to analyze various methodologies to further find the optimum quantitative assessment given objective data. To conclude, one cannot stress enough the fact that the assessment of technological level is one of the most important steps with regards to establishment of R&D plans and policies. Considering limitations of current assessments approaches (e.g., Delphi), our novel quantitative approach would provide a good alternative mean. In addition, it will be also interesting to combine both qualitative and quantitative assessments thereby providing unbiased results in future.

There are several limitations of the study. First, since the technology level is a type of intangible indicator thus one should not solely rely on assessment utilizing quantitative data. In addition to scientific literature and patents, both quantitative and qualitative data (e.g., human resources, infrastructure, and clinical trials) should be comprehensively employed for the assessment, if applicable. Second, in the present study, the ranking of technology levels were made based on relative values determined through the equation. To take an example, in case of the ACS, the technology level of US was found to be 849.3 whereas South Korea was 146.7 (Table 14). These values can be used to rank nations yet should not be interpreted as the US technology level is 5.8 fold higher than South Korea because these values are relative values. In addition, even though we deliberately selected the largest database (i.e., Scopus) to retrieve scientific publications, there are other databases (e.g., Science Citation Index) and available that may list journals other than we were able to obtain from the Scopus. Last, in the present study, three major stem research fields were subjected to the analysis thus, it would be interesting and informative to further investigate subspecialized stem cells (e.g., cancer stem cells, foetal stem cells, and dental stem cells) and their technological field through the proposed tool in future studies. Utilization of technology diffusion models (e.g., Bass model or Logistic model) for the prediction of stem cell research might be also interesting to examine in conjunction with current trends and outcomes herein (Rao and Kishore 2010).

## Abbreviations

ASC: adult stem cell
CA: Canada
CN: China
CHL: cited half-life
ESC: embryonic stem cell
EU: European union
FR: France
DE: Germany
hESC: human embryonic stem cell
iPSC: induced pluripotent stem cell
IL: Israel
JP: Japan
JST-CRDS: Japan Science and Technology Agency–Center for Research and Development Strategy
KISTEP: Korea Institute of Science and Technology Evaluation and Planning
OSTP: Office of Science and Technology Policy
PCA: principal component analysis
R&D: research and development
SG: Singapore
SSCC: Singapore Stem Cell Consortium
KR: South Korea
TW: Taiwan
UK: United Kingdom
US: United States
USPTO: United States Patents and Trademark Office
